# Meeting of the Maryland State Veterinary Medical Society

**Published:** 1893-01

**Authors:** A. W. Clement

**Affiliations:** Secretary pro tempore


*Meeting of the Maryland State Veterinary Medical Society.*—The regular monthly meeting of the Maryland State Veterinary Medical Society was held at the residence of Dr. Dougherty, on Monday evening, December 18th, the President, Dr. Martinet, in the chair. In the absence of the regular Secretary, Dr. Clement was appointed Secretary *pro tempore.* The following members were present: Drs. Barrow, Clement, Dougherty, Hoffman and Martinet.

The reading of the minutes of the previous meeting was dispensed with.

The Ambulance Committee reported through its Chairman, Dr. Dougherty.

Dr. Dougherty read the following paper on "Thermometry." Vide page 14.

After an interesting discussion, the meeting adjourned, with a vote of thanks to the essayist. A. W. CLEMENT, Secretary *pro tempore.*

